# The Effectiveness of Durian Peel as a Multi-Mycotoxin Adsorbent

**DOI:** 10.3390/toxins12020108

**Published:** 2020-02-08

**Authors:** Saowalak Adunphatcharaphon, Awanwee Petchkongkaew, Donato Greco, Vito D’Ascanio, Wonnop Visessanguan, Giuseppina Avantaggiato

**Affiliations:** 1 School of Food Science and Technology, Faculty of Science and Technology, Thammasat University, 99 Mhu 18, Paholyothin road, Khong Luang, Pathum Thani 12120, Thailand; s.adunphatcharaphon@hotmail.com (S.A.); awanwee@tu.ac.th (A.P.); 2 Institute of Sciences of Food Production (ISPA), National Research Council (CNR), Via Amendola 122/O, 70126 Bari, Italy; donato.greco@ispa.cnr.it (D.G.); vito.dascanio@ispa.cnr.it (V.D.); 3 Functional Ingredient and Food Innovation Research Group, National Center for Genetic Engineering and Biotechnology (BIOTEC), National Science and Technology Development Agency (NSTDA), 113 Thailand Science Park, Phahonyothin Road, Pathumthani 12120, Thailand; wonnop@biotec.or.th

**Keywords:** mycotoxins, durian peel, agricultural by-products, biosorption, gastrointestinal digestion model, decontamination, equilibrium isotherms

## Abstract

Durian peel (DP) is an agricultural waste that is widely used in dyes and for organic and inorganic pollutant adsorption. In this study, durian peel was acid-treated to enhance its mycotoxin adsorption efficacy. The acid-treated durian peel (ATDP) was assessed for simultaneous adsorption of aflatoxin B_1_ (AFB_1_), ochratoxin A (OTA), zearalenone (ZEA), deoxynivalenol (DON), and fumonisin B_1_ (FB_1_). The structure of the ATDP was also characterized by SEM–EDS, FT–IR, a zetasizer, and a surface-area analyzer. The results indicated that ATDP exhibited the highest mycotoxin adsorption towards AFB_1_ (98.4%), ZEA (98.4%), and OTA (97.3%), followed by FB_1_ (86.1%) and DON (2.0%). The pH significantly affected OTA and FB_1_ adsorption, whereas AFB_1_ and ZEA adsorption was not affected. Toxin adsorption by ATDP was dose-dependent and increased exponentially as the ATDP dosage increased. The maximum adsorption capacity (Q_max_), determined at pH 3 and pH 7, was 40.7 and 41.6 mmol kg^−1^ for AFB_1_, 15.4 and 17.3 mmol kg^−1^ for ZEA, 46.6 and 0.6 mmol kg^−1^ for OTA, and 28.9 and 0.1 mmol kg^−1^ for FB_1_, respectively. Interestingly, ATDP reduced the bioaccessibility of these mycotoxins after gastrointestinal digestion using an in vitro*,* validated, static model. The ATDP showed a more porous structure, with a larger surface area and a surface charge modification. These structural changes following acid treatment may explain the higher efficacy of ATDP in adsorbing mycotoxins. Hence, ATDP can be considered as a promising waste material for mycotoxin biosorption.

## 1. Introduction

Mycotoxins are fungi-derived metabolites capable of causing a dverse effects to both humans and animals. They are produced by toxigenic fungi, including *Aspergillus*, *Penicillium*, *Alternaria*, and *Fusarium* species, under specific temperature and humidity conditions [1,2,3,4]. The main mycotoxins occurring in food and feedstuffs are aflatoxins, ochratoxins, zearalenone, deoxynivalenol, and fumonisins [4,5]. Contamination by mycotoxins is common in primary agricultural commodities such as maize, rice, wheat, cereal products, meat, and dried fruits [5,6,7,8]. Multi-mycotoxin contamination of food and feedstuffs depends on environmental conditions and type of substrate [9]. A multi-mycotoxin-contaminated diet may induce acute mycotoxicosis with several chronic adverse effects, being mutagenic, carcinogenic, teratogenic, estrogenic, and immunosuppressive [10]. The combined consumption of different mycotoxins may produce synergistic toxic effects [9,11]. Mycotoxin consumption by livestock leads to economic losses for the feed industry and in international trade [12]. Since mycotoxin contamination cannot be completely prevented in pre-harvesting or post-harvesting, it is very difficult to avoid in agricultural commodities [5]. Decontamination strategies therefore play an important role in helping to reduce exposure to mycotoxin-contaminated feed. Strategies that have been developed for mycotoxin reduction in feedstuffs include physical, chemical, and biological methods. However, most have considerable limitations in practical applications [13]. The addition of mycotoxin binders (including activated charcoal, aluminosilicates, and agricultural wastes) to contaminated feed is an innovative and safe approach to counteracting the harmful effects of mycotoxins to livestock [12,14,15,16,17]. Mycotoxin adsorbents have several disadvantages, including the adsorption of essential nutrients and trace elements, as well as a rather narrow spectrum of action towards the pool of mycotoxins frequently found in feedstuffs. Therefore, it is very important to find new low-cost and biosustainable mycotoxin adsorbents that are able to simultaneously bind the main mycotoxins of zootechnical interest. Recently, the use of agricultural wastes as mycotoxin biosorbents has been investigated since they have a porous structure and contain a variety of functional groups, including carboxyl and hydroxyl groups, which may be involved in the binding mechanisms of mycotoxins [18,19]. In a recent study, [16] compared the ability of different agricultural by-products to adsorb mycotoxins from liquid media using the isotherm adsorption approach. Grape pomaces, artichoke wastes, and almond hulls were selected as the best mycotoxin biosorbents, being effective in adsorbing AFB_1_, ZEA, and OTA. Taking into account these findings, the present study evaluates the efficacy of durian peel waste as an additive for mycotoxin decontamination of feed. Durian Monthong (*Durio zibthinus*) is a popular fruit in Thailand and has many consumers. A large amount of durian peel is thrown away, resulting in social and environmental problems linked to waste disposal. As durian peel contains cellulose (47.2%), hemicellulose (9.63%), lignin (9.89%), and ash (4.20%), it has been extensively studied as a fuel and adsorbent of pollutants and heavy metals [20,21,22,23]. To the best of our knowledge, no research has reported reporting the use of durian peel as a multi-mycotoxin binder. The aim of this study is to assess the efficacy of durian peel as a binder, both untreated and acid-treated, in adsorbing the mycotoxins of major concern (aflatoxins, ochratoxins, zearalenone, deoxynivalenol, and fumonisins). The equilibrium isotherm approach was used to study mycotoxin reduction in liquid media at physiological pH values. In addition, the efficacy of these agricultural by-products in reducing mycotoxin bioaccessibility was assessed using a static, validated gastrointestinal model.

## 2. Results and Discussion

### 2.1. Characterization of Durian Peel

**Figure 1:**
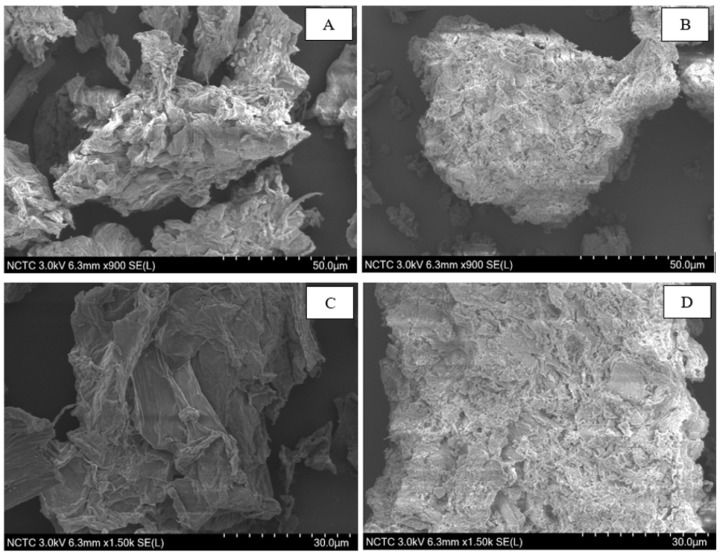
SEM images of durian peel (DP) and acid-treated durian peel (ATDP) at 900× and 1500× magnification. (**A**,**B**): DP and ATDP at 900×; (**C**,**D**): DP and ATDP at 1500×.

The surface morphology and elementary composition of DP and ATDP were determined using SEM–EDS. SEM images showed that acid treatment of DP had the effect of modifying its surface (Figure 1). More cavities were recorded on the surface of ATDP than DP. The study of Lazim et al. [22] reported more pores on a DP surface after treatment with sulfuric acid, providing a higher capacity in the removal of bisphenol A. These findings suggest that a change in the morphological structure of the DP surface following acid treatment may affect mycotoxin adsorption.

**Figure 2:**
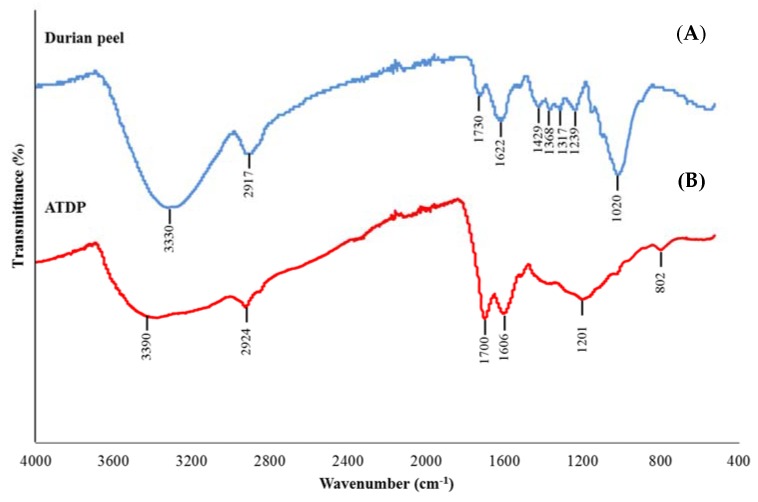
FT-IR spectra of DP (**A**) and ATDP (**B**).

EDS spectra analysis showed that C and O constitute the major elements of the materials, with C as the dominant component (data not shown). These results are in accordance with the study of Charoenvai [21], which classified the major components of DP as cellulose (47%), hemicellulose (10%), lignin (10%), and ash (4%). Acid treatment affects the elemental composition of the DP surface, thus increasing the proportion of C. The functional groups present on the DP and ATDP surfaces were identified by FTIR (Figure 2). The FTIR spectra of DP obtained were similar to those reported by Lazim et al. [19]. A first intense spectrum band was observed at 3330 cm^−1^, corresponding to O–H stretching vibrations and H bonding of cellulose, pectin, and lignin, which are the major fiber components of fruit peel [24,25]. A second peak was observed at 2917 cm^−1^, corresponding to C–H stretching vibrations of the methyl or methylene groups. Interestingly, no peak vibrations were found in the range at 2800–2300 cm^−1^, which represent N–H or C=O stretching vibrations of the amine and ketone functional groups. The peak at 1730 cm^−1^ corresponded to C=O stretching vibrations of the carbonyl group, while the peaks at 1622 cm^−1^ were related to the amide band (-CONH_2_). Peaks in the range 1500–1200 cm^−1^ were assigned to strong asymmetric carboxylic groups, methyl groups (bending vibration), aromatic amines, and C–O stretching vibrations of carboxylic acids [25]. Interestingly, a shift in all peak vibrations was observed in the FTIR spectra of ATDP and DP. In addition, ATDP produced no peaks in the range 1450–1250 cm^−1^. This suggests that modification by acid treatment affected the amine and methyl groups in the DP structure, resulting in a change in adsorption features. Ngabura et al. [25] observed that acidic groups, carboxyl, hydroxyl, and amides are involved in biosorption by DP. Zeta potential values for ATDP and DP differed substantially, with ATDP higher than DP. At pH 3, these values were −23.20 mV for ATDP and −2.55 mV for DP. This difference in zeta potential can be explained by modification of the DP structure, induced by acid treatment. In a previous study [25], acid treatment of DP affected the physical properties of the material. In our study, ATDP had greater BET pore volumes, pore diameters, and surface area (Table 1). These physical properties create greater adsorption at the surfaces. Ngabura et al. [25] found that hydrochloric acid-modified DP (HAMDP) had a more porous structure with a larger surface area than the pristine peel. The BET surface area is negatively correlated with the nanoparticle size, and the nanoparticle size of ATDP was 21-fold less than that of DP (Table 1). The same ratio was observed when comparing the surface areas of the ATDP and DP, with the surface area of ATDP being 21-fold higher than that of DP. This structural modification of the adsorbing surface following acid treatment was confirmed by the SEM–EDS images, which showed a more porous surface on the ATDP. The physico-chemical characterization suggested that the materials have different characteristics and are expected to differently in mycotoxin adsorption.

### 2.2. Screening of DP and ATDP as Multi-Mycotoxin Adsorbing Agents

DP and ATDP at 5 mg/mL dosage (0.5% *w/v*) were preliminarily tested for their ability to bind the mixture of five mycotoxins. Adsorption experiments were performed at a constant temperature of 37 °C and media of pH 3 and 7, using 1 mM citrate or 100 mM phosphate buffer. To measure mycotoxin adsorption by ATDP at pH 7, a 100-fold concentrated phosphate buffer was required since the ATDP suspension acidified the 1 mM phosphate buffer. As shown in Table 2, adsorption by DP and ATDP depended on the type of mycotoxin and pH of the medium. Maximum mycotoxin adsorption by DP was 53% for ZEA (pH 3), 46% for AFB_1_ (pH 3), and 18% for OTA (pH 3). AFB_1_ and ZEA adsorption was not affected by pH. OTA adsorption occurred mainly at pH 3, while FB_1_ and DON adsorption was negligible (≤2%). Interestingly, treatment with sulfuric acid significantly increased adsorption of most mycotoxins assayed in the study. The ATDP reduced AFB_1_ and ZEA by more than 98% in media at pH 3 and 7. OTA adsorption by ATDP at pH 3 and 7 was significantly higher than adsorption by DP, being 97% at acid pH and 42% at neutral pH. Acid treatment of DP also increased FB_1_ adsorption, but at acidic pH only. At pH 3, FB_1_ adsorption was 86%, while no adsorption was observed at pH 7. Acid treatment did not improve DON adsorption, which in all cases was less than 13%. As previously reported [19], treatment of DP with sulfuric acid modified the physico-chemical properties of the DP adsorption surface, increasing the binding sites available for mycotoxin adsorption.

### 2.3. Effect of Medium pH on Mycotoxin Adsorption and Desorption

**Figure 3:**
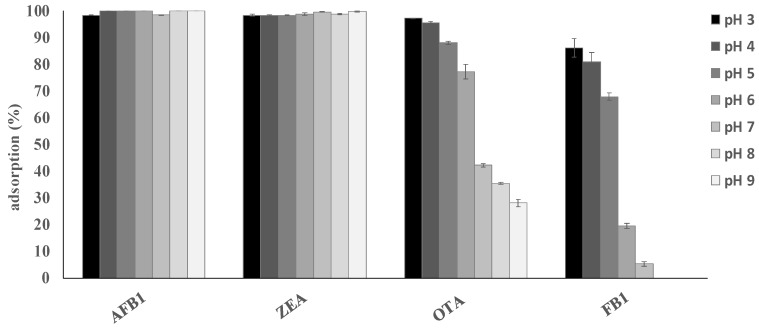
Effect of pH on AFB_1_, ZEA, OTA, and FB_1_ adsorption by ATDP tested at 5 mg/mL dosage towards a multi-mycotoxin solution containing 1 μg/mL of each toxin. Values are means of triplicate experiments.

Medium pH is an important parameter that affects the binding of mycotoxins by adsorbent materials, by affecting both the charge distribution on the surface of the adsorbents and the degree of ionization of the adsorbates. This is more important for adsorption processes in which electrostatic interactions are involved. An effective multi-mycotoxin adsorbent should sequester a large spectrum of mycotoxins with high efficacy, regardless of the medium pH, and should keep these contaminants bound along the compartments of a GI tract, where pH values ranging from 1.5–7.5 can be encountered. The results for pH (Figure 3) confirmed that AFB_1_ and ZEA adsorption by ATDP was stable within the GI tract of monogastric animals since 100% of the toxins were adsorbed at pH values ranging from 3 to 9. A desorption study was performed to assess whether a change of pH caused a release of the sequestered toxins. Mycotoxins were first adsorbed onto ATDP at pH 3, and then the pellet containing the adsorbed mycotoxins was washed first with a buffer at pH 7 and then with methanol. Washing solutions were analyzed for mycotoxin release. As shown in Table 3, AFB_1_ and ZEA adsorption was 100% at pH 3. No release was observed in the pH range from 3 to 7. The organic solvent (methanol) extracted 34% of the AFB_1_ and 85% of the ZEA, suggesting stronger binding of AFB_1_ by ATDP than by ZEA. OTA or FB_1_ adsorption and pH were inversely correlated. The adsorption efficacy of ATDP decreased as the pH increased (Figure 3). As OTA and FB_1_ hold acid groups in their structure, the pH of the medium is expected to affect the extent of mycotoxin adsorption [26]. OTA adsorption decreased from 97% to 28% as the pH was increased from 3 to 9. Similarly, FB_1_ was adsorbed mainly at pH 3, falling to 5% at pH above 6. However, despite the strong pH effect observed for OTA and FB_1_, ATDP was effective in retaining the adsorbed fractions after the medium pH was changed from 3 to 7 (Table 3). The organic solvent extracted half of the adsorbed OTA, while FB_1_ was poorly desorbed (7%). Overall, our study suggests that ATDP is highly efficacious in retaining FB_1_, AFB_1_, and OTA when a strong solvent is used. DP is an agricultural waste fiber. In addition to cellulose, hemicellulose, and lignin, it contains phenolic compounds with important biological properties [21]. The specific combination of these chemical components, and the increased adsorption surface obtained by acid treatment, explains the mycotoxin adsorption properties of ATDP.

### 2.4. Effect of ATDP Dosage

**Figure 4:**
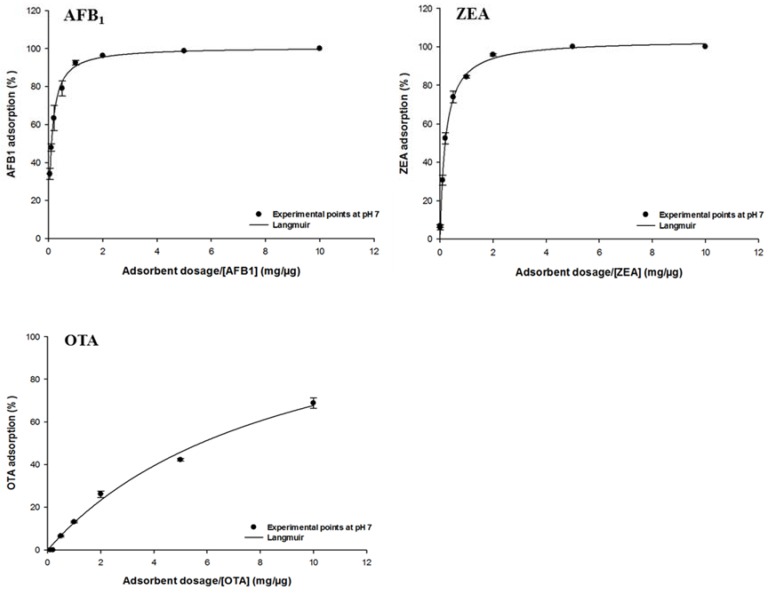
Effect of adsorbent dosage on AFB_1_, ZEA, and OTA adsorptions by ATDP. Equilibrium adsorption isotherms were obtained at constant temperature (37 °C) and at pH 7 by testing a fixed amount of toxin (1 μg/mL) with increasing adsorbent dosages (0.1–10 mg/mL).

The effect of ATDP dosage on mycotoxin adsorption was investigated using equilibrium adsorption isotherms. The goal was to calculate the optimal adsorbent dosage for further adsorption tests and to compare the efficacy of ATDP in simultaneously binding different mycotoxins. As shown in Figure 4, AFB_1_, ZEA, and OTA removal from a neutral medium increased as the dosage increased. Experimental values adsorption onto ATDP were in the ranges of 48–100% for AFB_1_, 31–100% for ZEA, and 0–69% for OTA. No significant FB_1_ adsorption was observed at pH 7. The adsorption plots for all toxins showed a characteristic L-shape (Figure 4) and were well fitted by the Langmuir model (R^2^ > 0.99). This model allowed calculation of both the theoretically estimated maximum adsorption Ads_max_ and the C_50_, which is the theoretically-estimated adsorbent dosage to achieve a 50% reduction of the absorbable toxin [26]. Predicted Ads_max_ values were 101 ± 1% for AFB_1_, 104 ± 1% for ZEA, and 103 ± 1% for OTA (Table 4). Additionally, C_50_ values listed in Table 4 suggest a higher efficacy of ATDP adsorption of AFB_1_ and ZEA than of OTA. It should not therefore be useful to increase the dosage of ATDP beyond 6 mg/mL when sequestering AFB_1_, ZEA, and OTA from a 1 µg/mL solution (Table 4). In the following equilibrium isotherm studies, the optimal adsorbent dosages were fixed in the range of 0.5 to 5 mg/mL depending on the toxin.

### 2.5. Equilibrium Adsorption Isotherm

**Figure 5:**
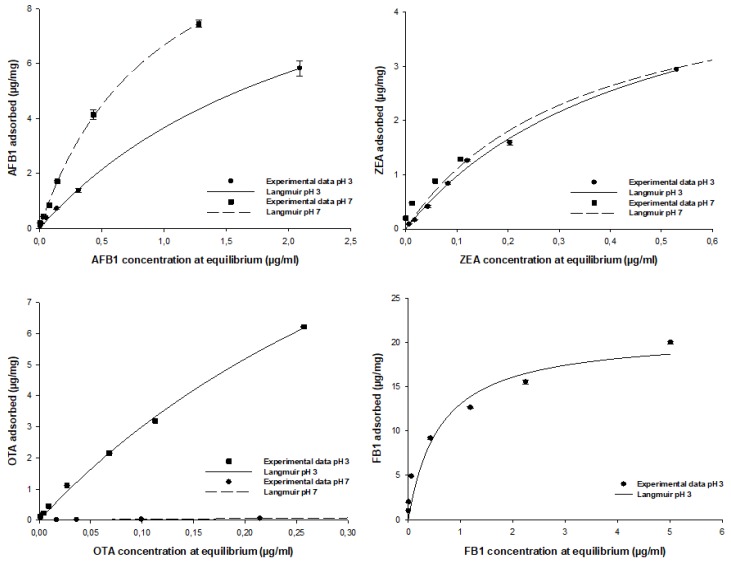
Effect of mycotoxin concentration on AFB_1_, ZEA, OTA, and FB_1_ adsorption by ATDP. Equilibrium adsorption isotherms were obtained at constant temperature (37 °C) and pH (3 and 7) by testing a fixed amount of ATDP with increasing toxin concentrations (0.025–7.5 μg/mL).

Isotherms are an effective approach to the study of surface adsorption mechanisms, surface properties, and an adsorbent affinity [16]. Nonlinear regression was used to assess the goodness of fit and to calculate the parameters involved in the adsorption mechanism (Ads_max_, K_L_). The mathematical models, including the Freundlich, Langmuir, and Sips equations, were used to predict the amount of AFB_1_, ZEA, OTA, and FB_1_ adsorbed by ATDP. The model that met regression analysis requirements (homogeneity of variance and normality assumptions), providing a lower statistical error, was used to fit the experimental data (Table 5). The amount of AFB_1_, ZEA, OTA, and FB_1_ adsorbed per unit mass of ATDP increased gradually as the mycotoxin concentration in the working solution increased. Isotherms showed an exponential relationship and a typical L (Langmuir) shape. In all cases, regardless of medium pH, the Langmuir model was found to best fit the experimental adsorption data. This model assumes that adsorption occurs at definite localized sites, which are identical and equivalent [27]. This implies that the adsorption of AFB_1_, ZEA, OTA, and FB_1_ by ATDP is homogeneous. As shown in Table 5, AFB_1_ adsorption by ATDP produced isotherms showing similar Ads_max_ values at pH 3 and 7. However, affinity was affected by the pH of the medium. The K_L_ Langmuir constant, which is related to adsorbent affinity, was 2.5-fold higher at pH 7 than pH 3 (Table 5). The increase of pH from 3 to 7 induced an increase in the K_L_ value from 0.4 ± 0.1 L/mg (125 000 ± 31.250 L/mol) to 1.0 ± 0.1 L/mg (312.500 ± 31 250 L/mol). This resulted in an increase in AFB_1_ adsorption affinity. Experimental values for AFB_1_ adsorption in percent varied in the ranges of 50–84% at pH 3 and 76–100% at pH 7. The predicted values for maximum adsorption capacity were 12.7 ± 0.9 μg/mg (40.7 ± 2.9 mmol/kg) at pH 3 and 13.0 ± 0.4 μg/mg (41.6 ± 1.3 mmol/kg) at pH 7 (Table 5). These values were in agreement with experimental results obtained at both pH values and were consistent with previous reports that AFB_1_ adsorption by agricultural by-products is not dependent on medium pH [16,26]. Compared with previous studies of agricultural by-products, ATDP showed higher AFB_1_ adsorption. The Langmuir isotherm was also found to be the best model when studying ZEA adsorption by ATDP (Figure 5 and Table 5). ZEA adsorption was not affected by the change in medium pH from 3 and 7). The experimental values for ZEA adsorption ranged from 70% to 86% at pH 3 and 54% to 100% at pH 7. The predicted maximum adsorption capacity was 5.5 ± 0.3 μg/mg (17.3 ± 0.9 mmol/kg) at pH 3 and 4.9 ± 0.3 μg/mg (15.4 ± 0.9 mmol/kg) at pH 7. The Langmuir K_L_ parameters were 2.2 ± 0.2 L/mg (700.637 ± 63.694 L/mol) at pH3 and 2.9 ± 0.6 L/mg (923.567 ± 191.082 L/mol) at pH 7. ZEA is a resorcyclic acid lactone and a hydrophobic compound [28]. It is a weak acid due to the presence of the diphenolic moiety and has a pKa of 7.62 [28]. At the pH values considered in this study (pH ≤ 7), it should be in protonated, nonionic form. ZEA adsorption by ATDP may involve hydrophobic interactions occurring at homogeneous adsorption sites with similar energy, as suggested by the Langmuir *K*_L_ parameter values. Unlike AFB_1_ and ZEA adsorption, OTA adsorption by ATDP was widely affected by pH. The experimental values for OTA adsorption were 47–96% at pH 3 and 46–65% at pH 7. Maximum adsorption capacities calculated at pH 3 and 7 were 18.8 ± 1.5 μg/mg (46.6 ± 3.7 mmol/kg) and 0.26 ± 0.02 μg/mg (0.64 ± 0.05 mmol/kg), respectively. K_L_ Langmuir values calculated were 1.90 ± 0.21 L/mg (766.129 ± 84.677 L/mol) at pH 3 and 1.30 ± 0.12 L/mg (524.193 ± 48.387 L/mol) at pH 7. As OTA is an ionizable molecule, a change in pH is expected to affect adsorption. The decrease in both Ads_max_ and *K*_L_ values for OTA adsorption at pH 7 may reflect by the presence of an anionic form of the toxin, producing repulsion between the OTA molecules and negative charges on the ATDP surface. In addition, these results suggest that hydrophobicity is implicated in OTA adsorption. Indeed, OTA was preferentially adsorbed at pH 3 when the uncharged form was predominant. At pH 7, OTA hydrophobicity decreased, affecting mycotoxin adsorption. In conclusion, OTA adsorption by ATDP may involve several mechanisms, including electrostatic forces and hydrophobic interactions, whose roles depend on the pH of the medium. As observed for OTA, FB_1_ adsorption was dependent first on pH, then on the degree of ionization of the molecules. FB_1_ adsorption was achieved at pH 3 only, since no adsorption was recorded at pH 7 (Figure 5). The experimental values for FB_1_ adsorption were in the range 67–100%. The predicted maximum adsorption capacity was 20.9 ± 1.2 μg/mg (28.9 ± 1.7 mmol/kg) at pH 3 (Table 5). The Langmuir K_L_ parameter was 1.7 ± 0.4 L/mg (1223.021 ± 287.769 L/mol). It can be concluded that, in acidic aqueous solutions, FB_1_ adsorption by ATDP is favoured and occurs mainly by polar non-covalent interactions. These include electrostatic interactions or hydrogen bonds involving the carboxylic functional groups. The efficacy of ATDP in removing mycotoxins from liquid media was significantly higher than previous reports of biosorbents: lactic acid bacteria, yeasts, moulds, and agricultural by-products [16,29,30,31,32,33,34].

### 2.6. Multi-Mycotoxin Adsorption in Simulated Gastrointestinal Fluid

The aim of this study is to assess whether ATDP, acting as a wide spectrum mycotoxin adsorbent, shows the same adsorption pattern after simulated gastrointestinal digestion. In vitro digestion models are successfully used as tools for assessing the bioaccessibility of nutrients and non-nutrients or the digestibility of macronutrients (e.g., lipids, proteins, and carbohydrates) in food matrices. These methods mimic physiological conditions in the gastrointestinal tract, taking into account the presence of digestive enzymes and their concentrations, pH, digestion time, and salt concentration, among other factors. In vitro assessment of mycotoxin bioaccessibility has been done through a number of approaches including static and dynamic digestion models, simulating the gastro-intestinal tract of monogastric animals and humans [35,36,37,38]. In the current study, the standardized digestion model described by Minekus et al. [39], comprising oral, gastric and small intestinal digestion phases, was used to assess the ability of ATDP to reduce the fraction of mycotoxins in the chyme available for absorption. For this purpose, a pool of mycotoxins containing AFB_1_, OTA, ZEA, and FB_1_ was subjected to gastro-intestinal digestion processes in the presence/absence of ATDP and, subsequently, the liquid fraction of the chyme obtained by centrifugation was analyzed for residual mycotoxin content. Mycotoxin bioaccessibility, calculated after gastric or intestinal (complete) digestion, was defined as the ratio between the initial mycotoxin content and the amount determined in the chyme at the end of the digestion phases. Under our experimental conditions, mycotoxin bioaccessibility was in the range 96.5–33.5% after gastric digestion and 96.3–39.7% after intestinal digestion (Table 6). For the mycotoxins tested, bioaccessibility decreased in the following order: AFB_1_ > FB_1_ > OTA > ZEA. It is worthy to note that the low values of ZEA or OTA bioaccessibility after gastrointestinal digestion were probably due to the formation of aggregates in the complex environment of the gastrointestinal digestive fluids. For the mycotoxins tested here, bioaccessibility at gastric and intestinal levels did not differ substantially. Digestion of ATDP in the presence of mycotoxin significantly reduced the fraction of toxins available for absorption, at both gastric and intestinal levels. Due to the inclusion of ATDP, mycotoxin bioaccessibility ranged from 25.8% to 0.8% at the gastric level, and from 78.9% to 4.3% at the intestinal level. These preliminary results suggest that ATDP was more effective in sequestering mycotoxins under the physiological conditions present in the stomach when the pH was low. Gastric digestion of ATDP reduced mycotoxin bioaccessibility by 93.6 ± 0.1% for AFB_1_, 97.6 ± 0.1% for ZEA, 96.2 ± 1.0% for OTA, and 67.3 ± 3.7% for FB_1_. At the completion of digestion, including the gastric and intestinal digestion phases, the reduction of AFB_1_ bioaccessibility by ATDP persisted (95.1 ± 0.1%). Smaller mycotoxin reductions were recorded at intestinal levels for ZEA (68.9 ± 1.1%), OTA (10.1 ± 2.6%), and FB_1_ (2.6 ± 2.7%). Overall findings suggest that AFB_1_, ZEA, OTA, and FB_1_ are quickly and efficiently sequestered by ATDP in the stomach. During the transit of the chyme from the stomach to the intestine, AFB_1_ and ZEA remained bound by ADTP, whereas some OTA and FB_1_ were released. The change in pH of the gastrointestinal fluids occurring during digestion may be the main factor driving OTA and FB_1_ release at the intestinal level. The model used in this study is a static one, and cannot simulate meal size, peristaltic movement, gastrointestinal transit, or absorption of digested products or water. However, it is a valid approach to investigating the adsorption/release of mycotoxins in a complex environment such as the stomach or the intestine.

## 3. Conclusions

Several studies have shown agricultural by-products to be suitable precursor materials for the effective and suitable removal of contaminants from aqueous media, including mycotoxins. Unfortunately, most of these biomaterials are unsuitable for adsorption in their raw form and must be pre-treated to improve their innate adsorption capacities. These pre-treatments include physical processes (drying, autoclaving, grinding, milling, or sieving) and chemical modification with reagents. Physico-chemical modification can enhance adsorption by reducing particle size and increasing surface area. In the present study, chemical activation of DP by sulfuric acid significantly improved surface area for adsorption, pore size distribution, and total pore volume. Structural characterization showed more cavities to be present on the surface of the ATDP than the untreated material (DP). C and O were the major surface elements. In addition, acid treatment changed the functional groups and charge on the adsorbent surface. These structural changes may explain the higher efficacy of ATDP than pristine DP in adsorbing mycotoxins. This is the first time that DP has been evaluated for its efficacy in sequestering mycotoxins. ATDP was found to be more effective than other mycotoxin biosorbent materials in removing mycotoxins from liquid mediums: lactic acid bacteria, yeasts, molds, or other agricultural by-products. Biosorption of mycotoxins was investigated by batch adsorption. Adsorption isotherms indicated that the process is dependent on key operating parameters, including medium pH, adsorbent dose, and initial mycotoxin concentration. Maximum adsorption capacities were described by the Langmuir isotherm. Values of Q_max_ determined at pH 3 and pH 7 were 40.7 and 41.6 mmol kg^−1^ for AFB_1_, 15.4 and 17.3 mmol/kg for ZEA, 46.6 and 0.6 mmol/kg for OTA, and 28.9 and 0.1 mmol/kg for FB_1_. DON was not sequestered by the raw or pre-treated agricultural by-product. The pH of the medium significantly affected OTA and FB_1_ adsorption, whereas AFB_1_ and ZEA adsorptions were not pH-dependent. As a consequence, digestion of ATDP (at 0.5% *w/v* dosage) in the presence of a multi-mycotoxin solution containing 1 µg/mL of each toxin (AFB_1_, OTA, ZEA, and FB_1_) by a static, validated gastrointestinal model, significantly reduced the bioaccessibility of all mycotoxins (>67% reduction) at gastric level. After digestion was completed, including a gastric and an intestinal step, significant reductions in mycotoxin bioaccessibility were recorded for AFB_1_ (94%) and ZEA (69%). These findings suggest that, during transit through the gastro-intestinal tract of a monogastric, most ingested AFB_1_ and ZEA can be adsorbed by ATDP and excreted in feces. In contrast, FB_1_ and OTA may be adsorbed in the stomach and released into the lumen of the intestine. Taking into account that most mycotoxins are quickly absorbed at the gastric level or in the upper part of the small intestine, the results of this study show the potential of ATDP as a multi-mycotoxin biosorbent. Further research is required to clarify the components of ATDP that are involved in the biosorption of mycotoxins and to confirm its efficacy in vivo.

## 4. Materials and Methods

### 4.1. Reagents and Samples

Solid mycotoxin standards (purity >98%), including aflatoxin B_1_ (AFB_1_), ochratoxin A (OTA), zearalenone (ZEA), deoxynivalenol (DON), and fumonisin B_1_ (FB_1_) were supplied by Sigma-Aldrich (Milan, Italy). All chemical reagents were purchased from Carlo Erba (Rouen, France), except for sodium chloride (NaCl) and potassium chloride (KCl), which were purchased from VWR (Leuven, Belgium). All solvents (HPLC grade) were purchased from J.T. Baker (Deventer, the Netherlands). Water was of Milli-Q quality (Millipore, Bedford, MA, USA). Digestive enzymes including α-amylase (from human saliva type IX-A, 1000–3000 U/mg), pepsin (from porcine gastric mucosa, 3200–4500 U/mg), pancreatin (from porcine pancreas, 4× USP), and bile bovine were supplied by Sigma-Aldrich (Milan, Italy). Mycotoxin adsorption studies were performed using different media (1 or 100 mmol/L) of different pH: citrate buffer at pH 3 (1 mM), acetate buffers at pH 4 and 5 (100 mM), and phosphate buffers at pH 6–9 (100 mM). Stock solutions of AFB_1_, OTA, ZEA, and DON (1 mg/mL) were prepared by dissolving solid commercial toxins in acetonitrile. FB_1_ was prepared in acetonitrile–water (50:50, *v/v*). Stock solutions were stored in the dark at 4 °C. A multi-mycotoxin stock solution containing 200 μg/mL of each toxin was prepared by mixing equal volumes of mycotoxin stock solutions. This was diluted with buffered solutions to prepare the mycotoxin working solutions for adsorption experiments. The Monthong durian peel (DP) used in the study was obtained from a local fruit shop in Bangkok, Thailand. The DP was washed with water to remove surface-adhered dirt and then cut into small pieces. These were oven-dried overnight, then, ground into fine powder using a mechanical grinder and passed through 35-mesh (0.5 mm) sieves. Particles smaller than 0.5 mm were collected and treated with sulfuric acid. The acid-treated material (ATDP) was heated overnight. It was then washed with distilled water to neutralize any acid residues and heated again. Untreated DP and ATDP, of the same particle size, were kept in a desiccator until use.

### 4.2. Physico-Chemical Characterization of DP and ATDP

The surface morphology and elementary composition of the DP and ATDP were investigated using scanning electron microscopy coupled with energy dispersive X-ray spectroscopy (SEM–EDS) (SU-5000, HITASHI, Tokyo, Japan). For identification of the chemical functional groups present on the DP and ATDP surfaces, a Fourier transform infrared spectroscopy (FTIR) (Nicolet 6700 FT-IR Spectrometer, Thermo Scientific, Waltham, MA, USA) analysis was performed in the spectral range from 4000 to 400 cm^−1^. The particle size and surface charge were measured using mastersizer and zetasizer instruments (Nano ZS, Malvern, UK). Brunauer–Emmett–Teller (BET) specific surface area, pore size distribution, and total pore volume were obtained from N_2_ adsorption–desorption isotherms using a surface area analyzer (Autosorb-1C, Quantachrome Corporation, Boynton Beach, FL, USA). Adsorption isotherms were obtained by measuring the amount of N_2_ adsorbed to the surface of both biosorbents at 77.26 K. Desorption isotherms were derived by removing the N_2_ adsorbed through a gradual pressure reduction. The methods used to characterize the DP and ATDP are reported elsewhere [25,40,41].

### 4.3. Multi-Mycotoxin Adsorption Experiments

DP and ATDP efficacy in adsorbing AFB_1_, OTA, ZEA, DON, and FB_1_ was evaluated at pH 3 and 7 using 1 mM citrate buffer and 100 mM phosphate buffer, respectively. Adsorption experiments were performed following the method of Avantaggiato et al. [26]. Briefly, DP and ATDP (< 500 μm particle size fraction) were weighed in a 4 mL silanized amber glass vial and suspended with an appropriate volume of multi-mycotoxin working solution buffered at pH 3 or 7. The suspensions were mixed for few seconds by vortex and then shaken for 90 min in a thermostatically-controlled shaker (KS 4000, IKA^®^-Werke GmbH & Co. KG, Staufen, Germany) at 37 °C and 250 rpm. After incubation, 1 mL of each suspension was transferred into an Eppendorf tube and centrifuged for 20 min at 18,000× *g* and 25 °C. Supernatant samples were analyzed for residual mycotoxin content following the HPLC and UPLC methods described by Avantaggiato et al. [26]. Adsorption experiments were carried out by adding 5 mg/mL of DP and ATDP to a multi-mycotoxin working solution comprising 1 μg/mL of each mycotoxin. To study the effect of pH on mycotoxin adsorption onto ATDP, independent experiments were performed in triplicate at pH values of 3–9, using a 5 mg/mL dosage (corresponding to 0.5% *w/v*). To investigate the desorption of mycotoxins from ATDP due to pH change, 5 mg of ATDP were dissolved with 1 mL of working solution at pH 3, containing 1 μg/mL of each toxin. Samples were incubated at 37 °C for 90 min in a rotary shaker (250 rpm). After centrifugation, the supernatants were completely removed and analyzed for residual mycotoxin content to calculate mycotoxin adsorption. The adsorbent pellets were washed with 1 mL of buffer at pH 7 and shaken for 30 min at 37 °C and 250 rpm. They were centrifuged, and the supernatants analyzed to assess mycotoxin desorption. This procedure was repeated by washing the pellet with 1 mL of methanol. Desorption studies were performed in triplicate. Adsorption (pH 3) and desorption (pH 7 and methanol) values were calculated for each toxin and expressed as percentages.

### 4.4. Equilibrium Adsorption Isotherms

Two sets of equilibrium adsorption isotherms were calculated to study the effect of adsorbent dosage and toxin concentration on simultaneous adsorption of AFB_1_, OTA, ZEA, and FB_1_. Due to the inefficacy of ATDP in adsorbing DON from all media used in the preliminary adsorption trials, DON was excluded from the study. In addition, since FB_1_ was not adsorbed at pH 7, equilibrium adsorption isotherms for FB_1_ were derived only at pH 3. Equilibrium adsorption isotherms matched to the experimental conditions (90 min equilibrium time, 37 °C, 250 rpm), as used for the preliminary adsorption experiments. The first set of adsorption isotherms was analyzed in triplicate, at constant pH 7, a fixed amount of toxin (1 μg/mL) with ATDP dosages ranging from 0.005–1% *w/v* (0.05–10 mg/mL) for AFB_1_, 0.005–1% *w/v* (0.005–10 mg/mL) for ZEA, and 0.01–1% *w/v* (0.1–10 mg/mL) for OTA. Adsorption data were expressed as a percentage of mycotoxin adsorbed and plotted as a function of ATDP dosage. Mycotoxin adsorption plots were fitted using non-linear regression models. The second set of isotherms was derived by testing a fixed amount of ATDP with buffered solutions at toxin concentrations from 0.025–15 μg/mL. These isotherms were used to calculate the parameters related to the adsorption process, including maximum adsorption capacity (Ads_max_) and affinity (K_L_). Adsorbent dosages were set from the preliminary adsorption experiments, using a 0.05% *w/v* (0.5 mg/mL) adsorbent dosage for AFB_1_, ZEA, and FB_1_. A 0.02% *w/v* (0.2 mg/mL) and a 0.5% *w/v* (5 mg/mL) dosage were used for OTA adsorption at pH 3 and 7, respectively. Adsorption isotherms were obtained by plotting the amount of mycotoxin adsorbed per unit of mass of adsorbent (Q_eq_) against the concentration of the toxin in the external phase (C_eq_) under equilibrium conditions, then fitting using non-linear regression models.

### 4.5. Simulated Gastrointestinal Digestion

**Figure 6:**
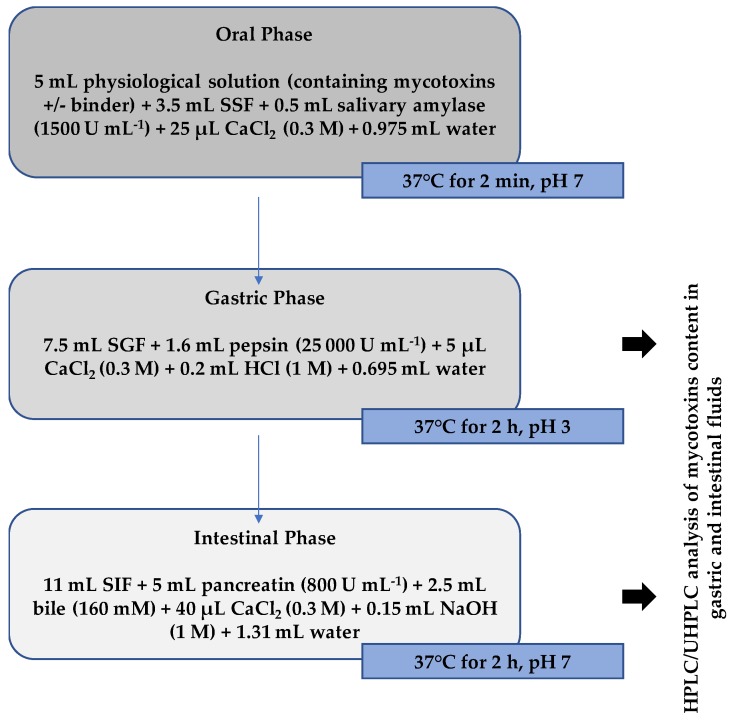
Flow diagram of the simulated digestion model proposed by Minekus et al. [39]. The model consists of a three-step procedure simulating the digestive processes in the mouth, stomach, and small intestine. SSF, SGF, and SIF stand for simulated salivary fluid, simulated gastric fluid, and simulated intestinal fluid, respectively

Since the gastrointestinal system is the primary target of mycotoxins, the “protective” effect of ATDP in reducing AFB_1_, OTA, ZEA, and FB_1_ bioaccessibility (i.e., the amount of mycotoxin that is released from the food matrix and is available for absorption through the gut wall) was determined by simulating a gastro-intestinal digestion process. In particular, mycotoxins in the presence or absence (negative controls) of ATDP were subjected to a simulated gastrointestinal digestion process and, subsequently, the digestive fluids obtained after gastric and/or intestinal digestion were analyzed for residual mycotoxin. The standardized digestion model described by Minekus et al. [39] was used in this study. This model describes a three-step procedure simulating digestive processes in the mouth, stomach, and small intestine (where most mycotoxin absorption takes place). A schematic representation of this model is presented in Figure 6. During simulated digestion, samples were rotated head-over-heels in a thermostatically controlled shaker (BFD53, ©BINDER-GmbH, Tuttlingen, Germany) at 37 °C for 2 min and 2 h to simulate, respectively, the oral phase and gastric or intestinal phases. Physiological and enzymatic solutions were prepared as described by Minekus et al. [39]. Simulated digestion started by mixing 5 mL of physiological solution, containing the multi-mycotoxin solution and ATDP at 5 mg/mL (0.5% *w/v*), with 3.5 mL of simulated salivary fluid (SSF). Next, 0.5 mL of salivary α-amylase SSF solution (1500 U/mL) was added, followed by 25 µL of CaCl_2_ (0.3 M) and 975 µL of water, and thoroughly mixed. The simulated gastric and intestinal solutions were then added in sequence. After 2 min incubation, simulated gastric juice at pH 3 was added, followed by 7.5 mL of simulated gastric fluid (SGF), 1.6 mL of porcine pepsin SGF solution (25,000 U/mL), 5 µL of CaCl_2_ (0.3 M), 0.2 mL of HCl (1 M), and 0.695 mL of water. After 2 h of simulated gastric digestion, intestinal fluids were added to the gastric chyme to mimic the digestion in the small intestine. Therefore, 20 mL of gastric chyme was mixed in sequence with 11 mL of simulated intestinal fluid (SIF), 5.0 mL of a pancreatin SIF solution (800 U/mL), 2.5 mL of bile (160 mM), 40 µL of CaCl_2_ (0.3 M), 0.15 mL of NaOH (1 M), and 1.31 mL of water. Before starting intestinal digestion, the pH was adjusted to 7. Two independent sets of simulated digestion experiments were performed to measure mycotoxin bioaccessibility at gastric and intestinal levels. The first set of trials was stopped after the gastric digestion phase. The second set included the gastric and intestinal phases. All experiments, including negative controls (without ATDP), were performed in quintuplicate. At the completion of digestion, the gastric or intestinal fluids were centrifuged at 4500 rpm for 15 min and analyzed by HPLC/UHPLC for residual mycotoxin content. Prior to LC analyses of AFB_1_, OTA, ZEA, and FB_1_, supernatant samples were cleaned up using immunoaffinity (IMA) columns provided by VICAM© (Watertown, MA, USA): AflaTest© WB, OchraTest© WB, ZearalaTest© WB, and FumoniTest© WB. Briefly, IMA columns were attached to a vacuum manifold (Visiprep™ SPE, Sigma Aldrich, Milan, Italy). Then, 500 µL of sample supernatants were passed through the columns at a flow rate of approximately one drop per second. Each column was washed with 5 mL of phosphate saline buffer (PBS) followed by 5 mL of water. AFB_1_, OTA, or ZEA were eluted by 2 mL of methanol in a 4 mL silanized amber vial. FB_1_ was eluted using 2 mL of methanol followed by 2 mL of water. Eluates were dried at 50 °C under an air stream (nitrogen was used for FB_1_) and the residues were re-dissolved with 250 μL of methanol/water (20:80, v/v), then vortexed for 1 min and injected into the LC systems. The LC analysis was performed following Greco et al. [16].

### 4.6. Data Calculation and Curve Fitting

Mycotoxin adsorption was measured from the difference between the amount of mycotoxin in the supernatant of the blank tubes and in the supernatant of the experimental tubes. The quantity present in the supernatant of the blank tubes was expressed as percentage of adsorption. ATDP was tested from two sets of equilibrium adsorption isotherms, using the methods reported by Avantaggiato et al. [26]. The first set of equilibrium adsorption isotherms was obtained by plotting the experimental adsorption data, expressed as percentage of mycotoxin adsorbed (Ads%), as a function of product dosage: Ads% = *f* (dosage). These data were transferred to SigmaPlot software (Systat.com, version 12.3) and fitted using the Langmuir isotherm model. The second set of adsorption isotherms was obtained by plotting the amount of mycotoxin adsorbed per unit of mass of product Q_eq_ against the concentration of the toxin in the external phase C_eq_, under equilibrium conditions: Q_eq_ = *f* (C_eq_). These data were transferred to SigmaPlot and fitted using different mathematical isotherm models (i.e., the Langmuir, Freundlich, and Sips models), as described by Avantaggiato et al. [26]. Mycotoxin bioaccessibility, expressed as a percentage, was calculated from the difference between the amount of mycotoxin found in the supernatant of gastric or intestinal fluid after each digestion process and the initial amount of toxin (mycotoxin intake). The efficacy of ATDP in reducing mycotoxin bioaccessibility was calculated as the difference between the bioaccessibility values measured after gastric or intestinal digestion of the control samples (with no ATDP) and experimental samples (containing the mycotoxin binder). Statistical analysis was performed by one-way analysis of variance (ANOVA) using the SigmaPlot software package. The Tukey–Kramer multiple-comparison post-hoc test was used, and differences were considered significant at *p* < 0.05.

## Figures and Tables

**Table 1:** BET single point method surface area analysis of DP and ATDP.

Adsorbent	Nanoparticle (nm)	Pore Volume (cm^3^/g)	Pore Diameter (nm)	Surface Area (m^2^/g)
DP	3032.45	0.004	7.22	1.98
ATDP	142.95	0.162	15.46	41.97

**Table 2:** Mycotoxin adsorptions by DP and ATDP tested at different pH values (7 and 3) and at 5 mg/mL of dosage towards a multi-mycotoxin solution containing 1 μg/mL of each toxin. Values are means of triplicate experiments ± standard deviations.

Toxin	DP		ATDP	
	pH 3	pH 7	pH 3	pH 7
AFB_1_	46 ± 4	37 ± 2	98.4 ± 0.1	98.4 ± 0.1
ZEA	53 ± 2	52 ± 4	98.4 ± 0.4	99.6 ± 0.2
OTA	18 ± 1	0.7 ± 0.6	97.3 ± 0.1	42.2 ± 0.2
FB_1_	0	2.3 ± 0.7	86 ± 3	0
DON	0	2 ± 1	2.0 ± 0.8	13 ± 2

**Table 3:** Mycotoxin adsorption and desorption from ATDP. Values are means ± standard deviations of triplicate independent experiments.

Toxin	Adsorption (%)	Desorption (%)	
	pH 3	pH 7	Methanol
**AFB_1_**	100	0	34 ± 3
**ZEA**	98.9 ± 0.4	0.8 ± 0.2	85 ± 4
**OTA**	99.0 ± 0.3	2.0 ± 0.5	48 ± 3
**FB_1_**	91 ± 3	1.6 ± 0.3	6.5 ± 0.5

**Table 4:** The theoretically estimated maximum adsorption (Ads_max_) and inclusion rate of ATDP to obtain a 50% reduction of the absorbable toxin (C_50_). Ads_max_ and C_50_ were calculated by fitting the data from Figure 4 with the Langmuir isotherm model.

Toxin	Ads_max_ (%)	C_50_ (mg/mL)
AFB_1_	101 ± 1	0.11
ZEA	104 ± 1	0.19
OTA	103 ± 1	5.77

**Table 5:** The isotherm model parameters of mycotoxin adsorption by ATDP calculated at different pH values.

	Parameters	AFB_1_		ZEA		OTA		FB1
		pH 3	pH 7	pH 3	pH 7	pH 3	pH 7	pH 3
Freundlich	K_f_ (±SE)	3.32 ± 0.05	6.43 ± 0.12	4.66 ± 0.17	3.33 ± 0.04	18.09 ± 0.17	0.17 ± 0.00	11.86 ± 0.14
	1/n (±SE)	0.76 ± 0.02	0.67 ± 0.03	0.69 ± 0.02	0.45 ± 0.01	0.79 ± 0.01	0.77 ± 0.02	0.33 ± 0.00
	R^2^	0.9977	0.9892	0.9850	0.9948	0.9997	0.9966	0.9976
	SS_res_	0.1705	1.2987	0.0120	0.1517	0.0272	0.0001	4.3605
	S_ylx_	0.1032	0.2849	0.1097	0.1041	0.0379	0.0022	0.4922
	PRESS	0.3656	1.6869	0.2661	0.1668	0.0336	0.0001	4.6588
	Normality	failed	failed	failed	passed	passed	passed	failed
	Constant Variance Test	passed	passed	passed	passed	passed	passed	failed
Langmuir	Ads_max_ (±SE)	12.69 ± 0.93	13.02 ± 0.40	5.47 ± 0.28	4.90 ± 0.34	18.82 ± 1.49	0.26 ± 0.02	20.89 ± 1.20
	K_L_ (±SE)	0.41 ± 0.05	1.05 ± 0.06	2.17 ± 0.18	2.91 ± 0.61	1.90 ± 0.21	1.30 ± 0.12	1.66 ± 0.35
	R^2^	0.9966	0.9980	0.9938	0.9566	0.9970	0.9974	0.9754
	SS_res_	0.2456	0.2394	0.0896	1.2691	0.2609	0.0001	43.4700
	S_ylx_	0.1239	0.1223	0.0706	0.3011	0.1170	0.0019	1.5540
	PRESS	0.4548	0.3329	0.111	1.9306	0.2996	0.0001	50.6400
	Normality	passed	passed	passed	passed	passed	passed	passed
	Constant Variance Test	passed	passed	passed	passed	passed	passed	passed
Sips	q_m_ (±SE)	-	11.67 ± 0.75	5.28 ± 0.73	-	-	-	91.70 ± 52.45
	A_s_ (±SE)	-	1.35 ± 0.90	2.38 ± 0.81	-	-	-	0.15 ± 0.10
	1/n = nH (±SE)	-	1.08 ± 0.97	1.02 ± 0.08	-	-	-	0.37 ± 0.03
	R^2^	-	0.9983	0.9938	-	-	-	0.9979
	SS_res_	-	0.206	0.0892	-	-	-	3.8247
	S_ylx_	-	0.1172	0.0724	-	-	-	0.4743
	PRESS	-	0.3403	0.1242	-	-	-	4.2181
	Normality		failed	passed	-	-	-	failed
	Constant Variance Test		passed	failed	-	-	-	failed

**Table 6:** Percentages of AFB_1_, ZEA, OTA, and FB_1_ recovered in the gastrointestinal fluids after simulated gastric or gastro-intestinal digestion processes. Mycotoxins were digested in the absence (control) or presence of ATDP. Values are means ± standard deviations of five independent experiments.

Toxin	Bioaccessibility (%)				Bioaccessibility Reduction (%)	
	Gastric Phase		Intestinal Phase		Gastric Phase	Intestinal Phase
	Control	+ATDP	Control	+ATDP		
AFB_1_	96.5 ± 0.9	6.3 ± 0.1	96.3 ± 0.8	4.3 ± 0.2	93.6 ± 0.1	95.1 ± 0.0
ZEA	33.5 ± 0.9	0.8 ± 0.1	39.7 ± 1.4	12.4 ± 0.4	97.6 ± 0.1	68.9 ± 1.1
OTA	49.6 ± 0.7	1.9 ± 0.5	43.6 ± 1.3	39.2 ± 1.1	96.2 ± 1.0	10.1 ± 2.6
FB_1_	78.8 ± 1.9	25.8 ± 2.9	81.2 ± 2.9	78.9 ± 1.9	67.3 ± 3.7	2.6 ± 2.7
